# Influenza Vaccination as Cardiovascular Prevention in Adults with Heart Disease

**DOI:** 10.3390/jcm15145343

**Published:** 2026-07-08

**Authors:** Clara Bonanad, Vivencio Barrios, Guillermo Barreres, Daniela Maidana, Esther Redondo

**Affiliations:** 1 Servicio de Cardiología, Hospital Clínico Universitario de Valencia, 46010 Valencia, Spain; 2 Fundación Para la Investigación del Hospital Clínico de la Comunidad Valenciana (INCLIVA), 46010 Valencia, Spain; gbarreres@incliva.es (G.B.); dmaidana@incliva.es (D.M.); 3 Centro de Investigación Biomédica en Red de Enfermedades Cardiovasculares (CIBER-CV), 28029 Madrid, Spain; 4 Servicio de Cardiología, Hospital Universitario Ramón y Cajal, Universidad de Alcalá, 28034 Madrid, Spain; vivenciobarrios@gmail.com; 5 Unidad Técnica de Vacunación y Salud Internacional, Organismo Autónomo Madrid Salud, 28007 Madrid, Spain

**Keywords:** influenza, influenza vaccination, cardiovascular prevention, cardiovascular disease, heart failure, myocardial infarction, older adults, high-dose vaccine

## Abstract

Seasonal influenza is not only a respiratory infection but also a clinically relevant trigger of acute cardiovascular events. In adults with established cardiovascular disease, particularly older adults and patients with recent acute coronary syndrome or heart failure, influenza vaccination should be considered a low-risk, evidence-supported component of cardiovascular prevention rather than solely protection against respiratory disease. The evidence addresses three related but distinct questions: influenza infection as a cardiovascular trigger; influenza vaccination versus placebo or no vaccination; and enhanced or high-dose vaccination versus standard-dose vaccination. Randomized trials and meta-analyses support reductions in major adverse cardiovascular events, cardiovascular mortality, and all-cause mortality in higher-risk secondary-prevention populations, with particularly persuasive evidence after myocardial infarction. Recent pragmatic active-comparator trials of high-dose inactivated influenza vaccine, including DANFLU-2 and GALFLU, and the individual-level pooled FLUNITY-HD analysis provide incremental evidence in adults aged 65 years or older, strongest for hospitalization for influenza or pneumonia, cardiorespiratory hospitalization, and heart failure hospitalization. The current priority is implementation: screening, offering, documenting, and communicating vaccination across hospitalization, outpatient cardiology, cardiac rehabilitation, heart failure pathways, primary care, pharmacies, and long-term care. Influenza vaccination should complement, not replace, established guideline-directed cardiovascular therapies.

## 1. Introduction

In cardiovascular medicine, residual risk is usually discussed in terms of lipids, thrombosis, blood pressure, glycemic control, or heart failure progression. However, a recurrent respiratory infection such as influenza also modifies cardiovascular risk in a clinically meaningful way. The 2025 European Society of Cardiology clinical consensus statement and the 2025 American College of Cardiology guidance both position vaccination within modern cardiovascular prevention, while the World Health Organization continues to recommend annual influenza vaccination for older adults and people with chronic health conditions [1,2,3,4,5].

Influenza is associated with excess morbidity and mortality well beyond the respiratory tract. Elderly or frail patients and people with chronic cardiovascular disease are particularly vulnerable to complications such as pneumonia, functional decline, myocardial infarction, stroke, arrhythmias, and heart failure decompensation [4,6]. This interaction is biologically plausible: influenza can amplify systemic inflammation, platelet activation, endothelial dysfunction, plaque instability, and oxygen supply-demand imbalance, thereby increasing the likelihood of acute cardiovascular events [6,7,8,9,10,11,12].

Against this background, influenza vaccination should be understood not only as infection prevention but also as a practical, low-risk measure that may reduce infection-related cardiovascular destabilization in selected high-risk populations.

## 2. Objectives and Scope

The aims of this narrative review are fourfold: first, to summarize the evidence linking influenza infection to acute cardiovascular destabilization; second, to review data on influenza vaccination versus placebo or no vaccination in adults with and without established cardiovascular disease; third, to discuss recent active-comparator evidence for high-dose or enhanced influenza vaccines, with special attention to DANFLU-2, GALFLU, FLUNITY-HD, and PANDA II; and fourth, to translate the evidence into practical implementation priorities for cardiology, primary care, pharmacies, long-term care, and public health. Compared with prior reviews, the intended contribution is an updated, clinically oriented synthesis that explicitly separates biologic and epidemiologic evidence, vaccine-versus-control evidence, and high-dose-versus-standard-dose evidence, and then connects these strands to implementable cardiovascular prevention pathways.

## 3. Evidence Selection and Conceptual Framework

Because this article is a narrative review rather than a systematic review or meta-analysis, the evidence-selection strategy was designed to identify the most clinically relevant data rather than to exhaustively pool effect estimates. Targeted searches were performed in PubMed/MEDLINE, ClinicalTrials.gov, and guideline or position-statement sources from the World Health Organization, European Society of Cardiology, American College of Cardiology, Spanish Ministry of Health, and SEMERGEN from database inception to May 2026. Search terms included influenza, influenza vaccination, cardiovascular disease, myocardial infarction, acute coronary syndrome, heart failure, stroke, major adverse cardiovascular events, high-dose influenza vaccine, standard-dose influenza vaccine, DANFLU-2, GALFLU, FLUNITY-HD, PANDA II, and INVESTED.

We prioritized randomized clinical trials, pragmatic randomized effectiveness studies, prespecified pooled analyses, systematic reviews, meta-analyses, and contemporary guideline or consensus statements. Observational studies were used mainly to characterize influenza infection as a cardiovascular trigger and to describe implementation gaps. English- and Spanish-language documents were considered. No formal risk-of-bias grading, duplicate screening, or quantitative synthesis was undertaken; therefore, effect estimates are presented descriptively and should be interpreted according to study design, comparator, and baseline population risk.

## 4. Benefits of Influenza Virus Vaccination in the General Population

Influenza should no longer be viewed as a purely respiratory event. Historical time-series analyses and contemporary epidemiologic studies have shown that periods of greater influenza circulation are accompanied by a rise in cardiovascular mortality and hospitalization, especially in older adults [4,6,7,8,9,10,11,12]. This temporal association is particularly striking for acute myocardial infarction: in a self-controlled study of laboratory-confirmed influenza, the risk of myocardial infarction increased approximately sixfold during the first week after infection [6]. In hospitalized adults with influenza, cardiovascular complications are also frequent; in one large cross-sectional study, approximately 12% developed acute cardiovascular events such as acute coronary syndrome or heart failure during admission [7]. At community level, greater influenza-like illness activity has also been associated with increased hospitalizations for heart failure [8].

**Figure 1:**
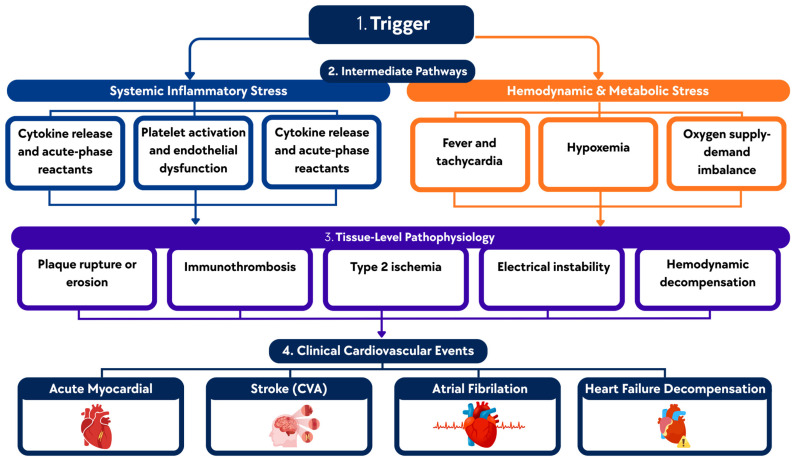
How influenza precipitates cardiovascular events. Abbreviations: CVA, Cerebrovascular Accident.

**Figure 2:**
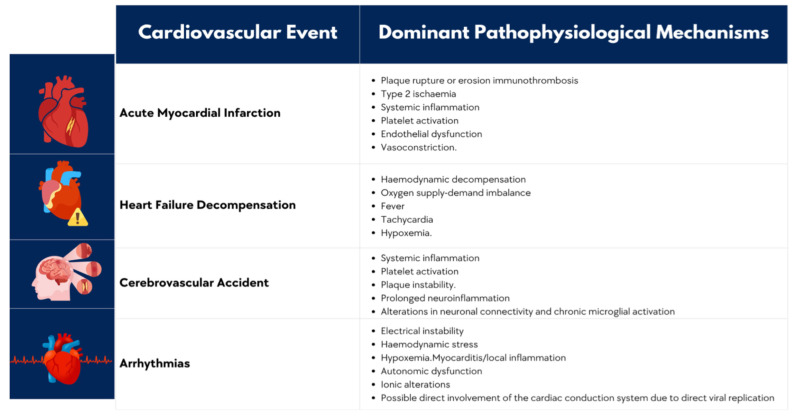
Cardiovascular complications of influenza and dominant mechanisms.

The mechanisms underlying this relationship are multifactorial and synergistic. Influenza infection can trigger cytokine release, acute-phase reactants, platelet activation, endothelial dysfunction, vasoconstriction, fever, tachycardia, and hypoxemia, thereby favoring plaque rupture or erosion, immunothrombosis, type 2 ischemia, electrical instability, and hemodynamic decompensation [10,11,12]. These processes provide a biological framework linking influenza infection with acute cardiovascular destabilization, including myocardial ischemic events, stroke, arrhythmias, and heart failure decompensation (Figure 1). The dominant mechanisms associated with each major cardiovascular manifestation are summarized in Figure 2.

**Figure 3:**
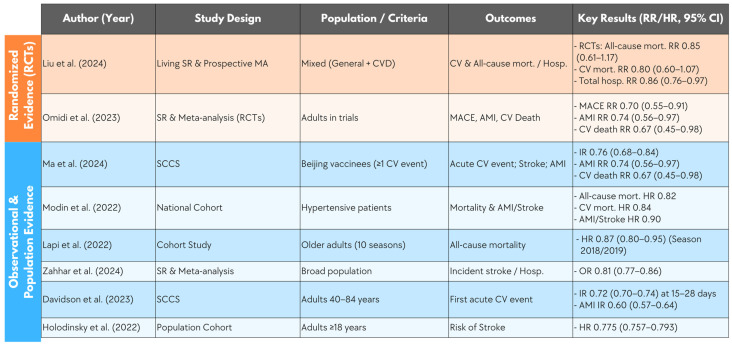
Cardiovascular evidence on the association between influenza vaccination and cardiovascular outcomes in the general population. Abbreviations: AMI, acute myocardial infarction; CV, cardiovascular; CVD, cardiovascular disease; HR, hazard ratio; IR, incidence ratio; MA, meta-analysis; MACE, major adverse cardiovascular events; OR, odds ratio; RCT, randomized clinical trial; RR, relative risk; SCCS, self-controlled case series; SR, systematic review.

In the general population, randomized trial evidence for major cardiovascular end points after influenza vaccination is less decisive than in secondary-prevention cohorts (Figure 3), probably because event rates are lower and trials were not originally powered for cardiovascular outcomes. Even so, the direction of effect is generally favorable. A living systematic review and prospective meta-analysis found that randomized evidence did not show statistically significant reductions in cardiovascular mortality or cardiovascular hospitalization in broad populations, but there was a reduction in all-cause hospitalization [13]. Large observational and self-controlled studies have added a complementary signal: influenza vaccination has been associated with fewer first acute cardiovascular events after vaccination, lower risk of myocardial infarction in the early post-vaccination period, and lower risk of stroke [14,15].

Taken together, the general-population evidence does two things. First, it establishes influenza infection as a clinically meaningful cardiovascular trigger. Second, it suggests that influenza vaccination likely confers a cardiovascular benefit at population level, even if the most robust and clinically important effects are concentrated in those with pre-existing cardiovascular disease or in older adults at higher baseline risk.

## 5. Specific Benefits of Influenza Virus Vaccination in People with Cardiovascular Disease

In people with established cardiovascular disease, the evidence is more consistent and more clinically persuasive than in lower-risk general-population samples. Across systematic reviews and meta-analyses, influenza vaccination has been associated with reductions in all-cause mortality, cardiovascular mortality, and major adverse cardiovascular events (MACE), particularly in populations with ischemic heart disease, acute coronary syndromes, or heart failure [16,17,18,19,20,21,22]. In the 2022 meta-analysis by Behrouzi and colleagues, vaccination was associated with a relative risk reduction of approximately 25% for all-cause mortality, 18% for cardiovascular mortality, and 34% for MACE in patients with and without cardiovascular disease, with particularly relevant effects in those at higher cardiovascular risk [17]. Other meta-analyses focused on coronary disease or acute coronary syndromes have reported similar directional findings, including lower mortality and lower MACE [18,19,21,22].

The signal is reinforced by randomized evidence. The IAMI trial showed that influenza vaccination administered before discharge after myocardial infarction or coronary intervention reduced the primary composite endpoint and was associated with a marked reduction in cardiovascular mortality at one year [23]. Earlier trials, including Phrommintikul et al. and FLUVACS, had already suggested cardiovascular benefit in acute coronary populations, albeit with smaller sample sizes [24,25]. The more recent meta-analysis by Modin et al., focused on patients with ischemic heart disease or heart failure, reported a 26% reduction in composite cardiovascular events, a 37% reduction in cardiovascular mortality, and a 28% reduction in all-cause mortality [20].

Heart failure deserves particular emphasis. In this phenotype, respiratory infection is a common and powerful trigger of decompensation. A meta-analysis in patients with heart failure found a significant reduction in all-cause mortality associated with influenza vaccination [26]. In addition, the PANDA II trial showed that vaccination before discharge in patients admitted with acute heart failure reduced the combined risk of death or readmission at 12 months [27]. This is highly relevant for cardiology practice, as it shifts vaccination from a generic recommendation to a specific in-hospital preventive measure.

**Figure 4:**
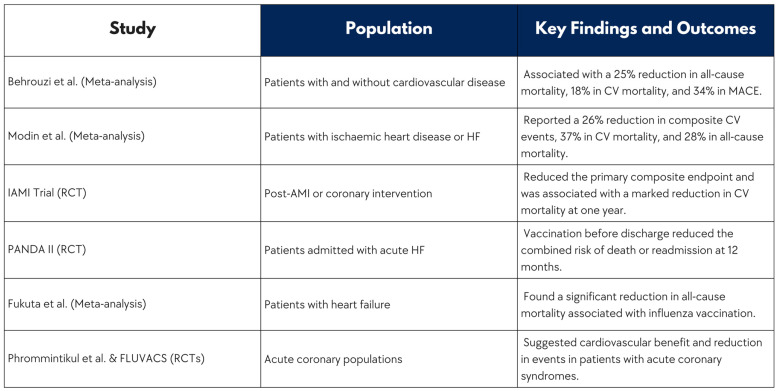
Clinical evidence for influenza vaccination versus placebo or no vaccination for cardiovascular outcomes. Abbreviations: AMI, acute myocardial infarction; CV, cardiovascular; MACE, major adverse cardiovascular events; RCT, randomized clinical trial.

Overall, the evidence in established cardiovascular disease supports a clear practical conclusion: annual influenza vaccination should be integrated into routine secondary prevention as a low-risk, evidence-supported preventive measure. This does not mean that vaccination is equivalent to statins, antithrombotic therapy, antihypertensive treatment, or guideline-directed heart failure therapy; rather, it complements those interventions by reducing infection-related cardiovascular destabilization and by closing a preventable-risk gap that is often missed during cardiovascular care. The main studies supporting the cardiovascular benefits of influenza vaccination are summarized in Figure 4 and Table 1.

## 6. Relevant Guidelines in This Area

Current guidance landscape is aligned in the same direction. The World Health Organization recommends annual influenza vaccination for target groups at higher risk of severe disease, including older adults and people with chronic health conditions [3]. The 2025 European Society of Cardiology consensus statement is particularly important for cardiology given that it explicitly frames vaccination as a new form of cardiovascular prevention rather than a peripheral topic delegated entirely to infectious diseases or public health [1]. Likewise, the 2025 American College of Cardiology guidance places adult immunization within cardiovascular care pathways and highlights opportunistic delivery in cardiology practice, especially during hospitalization and follow-up in high-risk patients [2].

In Spain, the 2024 adult vaccination recommendations coordinated by SEMERGEN (Sociedad Española de Médicos de Atención Primaria) also emphasize the role of enhanced-immunogenicity influenza formulations in older adults and provide a practical bridge between cardiology, primary care, and preventive care [5]. What remains suboptimal is not the consistency of recommendations, but their implementation. Vaccination coverage among patients with cardiovascular disease remains insufficient in many settings, and missed opportunities are common during clinic visits, rehabilitation programs, and hospital discharge transitions care [34]. Table 2 summarizes the main recommendations from these guidelines.

## 7. Efficacy and Safety of the High-Dose Flu Vaccine in the General Population: Recent Lessons from DANFLU-2, GALFLU, and FLUNITY-HD

The question is no longer only whether influenza vaccination is beneficial, but whether more immunogenic formulations can provide additional benefit in older adults. The biologic rationale is strong: immunosenescence, frailty, and multimorbidity reduce immune response to standard-dose vaccines, whereas enhanced influenza vaccines, including high-dose inactivated, adjuvanted standard-dose, and recombinant vaccines, contain larger antigen payloads or immune-enhancing components that provide higher immunogenicity compared to standard-dose formulations. High-dose inactivated influenza vaccine has demonstrated superiority against laboratory-confirmed influenza in adults aged 65 years or older, while recombinant influenza vaccine has shown similar superiority in adults aged 50 years or older, suggesting that these enhanced vaccine platforms may offer additional clinical value in populations with compromised immune responses [35,36].

Additional comparative efficacy data is expected to be available soon, notably, a randomized trial directly comparing the adjuvanted influenza vaccine with the standard dose vaccine against laboratory confirmed influenza in adults ≥ 65 years of age. (ClinicalTrials.gov. NCT05559723). Phase 3 randomized study of MF59-adjuvanted quadrivalent influenza vaccine vs. non-adjuvanted vaccine in adults ≥ 65 years [37].

The defining major clinical advance in 2025 came from two pragmatic randomized effectiveness trials embedded in routine care. DANFLU-2, conducted in Denmark, included 332,438 adults aged 65 years or older and did not meet statistical significance for its primary endpoint of hospitalization for influenza or pneumonia, although the point estimate favoured high-dose vaccine [29]. Importantly, however, the high-dose formulation reduced hospitalization for influenza and showed favourable signals for cardiorespiratory hospitalization, while maintaining a safety profile similar to that of the standard dose [29].

GALFLU, conducted in Galicia with 133,882 randomized participants aged 65 to 79 years, showed a statistically significant reduction in the primary endpoint of hospitalization for influenza or pneumonia, along with fewer influenza hospitalizations and cardiorespiratory hospitalizations, again without meaningful safety concerns [31]. When both trials were pooled in the prespecified FLUNITY-HD individual-level analysis, high-dose vaccination showed a statistically significant relative benefit for hospitalization for influenza or pneumonia and also reduced cardiorespiratory hospitalization and hospitalization for any cause [32].

**Figure 5:**
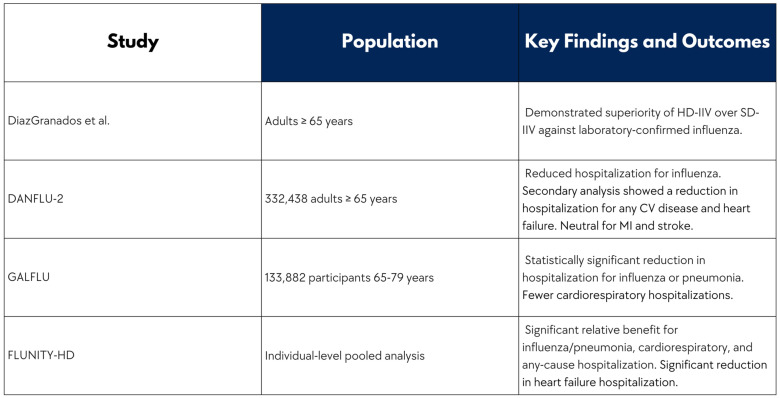
High-dose inactivated influenza vaccine (HD-IIV) versus standard dose (SD-IIV): key studies. Abbreviations: CV, cardiovascular.

**Figure 6:**
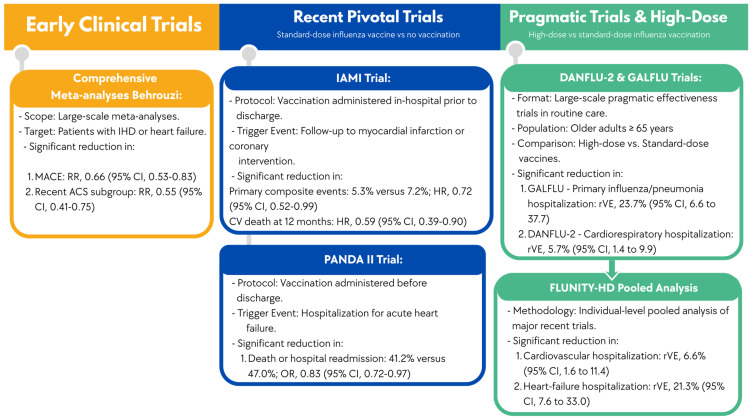
From respiratory vaccine to cardiovascular therapy: chronology of the evidence. Original figure developed from [16,17,18,19,20,21,22,23,24,25,26,27,28,29,30,31,32,33,34,35,36,37]. Abbreviations: ACS, acute coronary syndrome; CI, confidence interval; CV, cardiovascular; HR, hazard ratio; IHD, ischemic heart disease; MACE, major adverse cardiovascular events; OR, odds ratio; RR, relative risk; rVE, relative vaccine effectiveness.

These findings are clinically important for two reasons. First, they show that active-comparator vaccination trials can detect meaningful differences in severe outcomes at scale. Second, they suggest that in older adults at higher baseline risk, high-dose influenza vaccine can provide incremental protection on top of standard-dose vaccination. Figure 5 and Figure 6 summarize the main trajectory of this evidence. The evolution of evidence from early cardiovascular trials to recent pragmatic high-dose vaccine studies is shown in Figure 6.

## 8. High-Dose Vaccine in Patients with Cardiovascular Disease and for Cardiovascular Events

The cardiovascular subanalyses of DANFLU-2 and FLUNITY-HD are especially relevant because they address incremental cardiovascular outcomes among recipients of active influenza vaccination. In the prespecified secondary analysis of DANFLU-2, high-dose vaccine reduced hospitalization for any cardiovascular disease and showed the clearest signal for heart failure hospitalization, whereas no statistically significant superiority was demonstrated for myocardial infarction, stroke, cardiovascular death, or the overall MACE composite [30].

These neutral findings for atherothrombotic end points should be interpreted with caution. DANFLU-2 compared high-dose with standard-dose vaccine, not vaccination versus no vaccination. It therefore quantified incremental benefit beyond an already active comparator. The lack of statistical superiority for myocardial infarction or stroke does not imply absence of intrinsic cardiovascular benefit from influenza vaccination itself; rather, it means that the available active-comparator data do not justify claiming high-dose superiority for these specific end points. In addition, these end points are heterogeneous, and only a subset of events is likely to be directly attributable to attenuation of infection-related inflammatory and hemodynamic stress.

The pooled FLUNITY-HD cardiovascular analysis strengthened the overall hospitalization signal. Compared with standard-dose, high-dose vaccination reduced hospitalization for any cardiorespiratory disease and any cardiovascular disease, with the most robust and clinically compelling effect seen in hospitalization for heart failure [29]. In patients with pre-existing cardiovascular disease, the relative effects remained directionally consistent, and there was no convincing evidence of harmful trade-offs or clinically important safety concerns. This matters because even modest relative effects may translate into greater absolute event reductions in populations with higher baseline risk, such as those with atherosclerotic cardiovascular disease or chronic heart failure.

**Figure 7:**
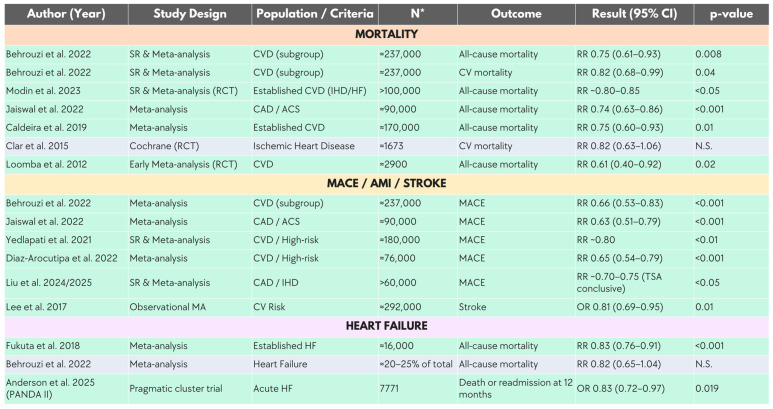
Evidence of the benefits of flu vaccination in patients with cardiovascular disease. Abbreviations: ACS, acute coronary syndrome; CAD, coronary artery disease; CI, confidence interval; CV, cardiovascular; CVD, cardiovascular disease; HF, heart failure; IHD, ischemic heart disease; MACE, major adverse cardiovascular events; N*, number of patients; N.S., non-significant; OR, odds ratio; RCT, randomized controlled trial; RR, relative risk; SR, systematic review; TSA, trial sequential analysis.

For clinicians, the practical take-home message is straightforward. The strongest incremental evidence for high-dose over standard-dose influenza vaccination currently lies in severe hospitalization outcomes, especially hospitalization for influenza or pneumonia, cardiorespiratory hospitalization, and heart failure hospitalization. This does not replace the broader evidence base supporting influenza vaccination in all eligible cardiovascular patients; rather, it helps refine prioritization within older and more vulnerable groups. Figure 7 provides a comprehensive synthesis of the clinical evidence supporting the cardiovascular benefits of influenza vaccination across different patient profiles and outcomes.

## 9. Next Steps and Future Directions: Time for Implementation

The value of a consensus document or narrative review ultimately depends ultimately on whether it changes care. In cardiovascular patients, influenza vaccination should move from abstract recommendation to operational pathway. This means verifying vaccination status at every relevant point of contact, offering vaccination opportunistically when possible, documenting the action, and ensuring communication across cardiology, primary care, pharmacy, rehabilitation, nursing homes, and public health services [1,2,5]. Table 3 translates this approach into practical settings.

Fragmentation between levels of care should be reduced through shared protocols, especially for patients with heart failure, coronary disease, chronic kidney disease, diabetes, or advanced age. Seasonal influenza vaccination should be systematically incorporated into the care pathways of heart failure clinics, cardiac rehabilitation units, and discharge checklists after myocardial infarction or decompensated heart failure.

In adults aged 60 years or older, high-dose influenza vaccination should be considered based on current available evidence, taking into account national or regional recommendations, local policies, and product availability. In parallel, vaccination strategies in this population should be supported by indicators not only of implementation, such as vaccine coverage, but also of relevant clinical outcomes, including hospitalization for influenza or pneumonia, cardiorespiratory hospitalization, cardiovascular hospitalization, heart failure readmission, and mortality. These metrics may help inform benchmarking and quality-improvement initiatives across centres and territories.

Other adult vaccines should complement but not eclipse the central role of influenza vaccination in this population. In practical terms, rather than recommending vaccination in general, the best cardiology strategy is to verify, vaccinate, document, and close the care loop.

## 10. Limitations of the Available Evidence

The literature combining vaccination and cardiovascular prevention is heterogeneous. Comparisons include vaccine versus placebo or no vaccination, high-dose versus standard-dose formulations, randomized trials, pragmatic effectiveness studies, and observational analyses. These designs answer different questions and should not be interpreted as if they were numerically interchangeable.

Observational evidence is vulnerable to confounding, residual confounding, confounding by indication, and healthy-user bias. Vaccinated individuals may differ from unvaccinated individuals in health-seeking behavior, access to care, socioeconomic status, adherence to cardiovascular medication, and baseline frailty. Although self-controlled designs and randomized trials mitigate some of these limitations, no single design fully resolves all sources of bias.

The magnitude of benefit is also context dependent. Influenza intensity, vaccine effectiveness in a given season, timing of vaccination, product availability, baseline cardiovascular risk, frailty, comorbidity burden, and outcome ascertainment all influence absolute and relative effects. Some randomized trials were underpowered for cardiovascular end points, terminated early, or relied on secondary or exploratory analyses; therefore, confidence intervals and multiplicity should be considered when interpreting subgroup or end-point-specific results.

Finally, the incremental evidence for high-dose vaccination comes mainly from older-adult populations and active-comparator trials. These data should not be extrapolated uncritically to younger cardiovascular cohorts or interpreted as proof of superiority for all cardiovascular outcomes. The strongest high-dose signal currently relates to severe respiratory, cardiorespiratory, and heart failure hospitalization outcomes, while evidence for myocardial infarction, stroke, cardiovascular death, and overall MACE superiority remains neutral or insufficient.

## 11. Conclusions

Influenza vaccination should be regarded as a low-risk, evidence-supported component of cardiovascular prevention intervention in adults with heart disease. The most convincing data for direct cardiovascular benefit come from randomized trials and meta-analyses in high-risk patients and in secondary prevention, where reductions in major adverse cardiovascular events, cardiovascular mortality, and all-cause mortality have been observed, especially after acute coronary syndrome or myocardial infarction.

In older adults, the high-dose inactivated influenza vaccine currently provides the strongest incremental evidence over standard dose for severe outcomes, particularly hospitalization for influenza or pneumonia, cardiorespiratory hospitalization, and heart failure hospitalization. The present evidence does not justify overclaiming superiority for myocardial infarction, stroke, cardiovascular death, or overall MACE in active-comparator trials, but it does strongly support the concept that preventing influenza and severe influenza is part of preventing cardiovascular destabilization.

The next step is implementation. In everyday practice, influenza vaccination belongs in cardiovascular prevention pathways before discharge, during outpatient follow-up, in rehabilitation, and in coordination with primary care, pharmacy, long-term care, and public health. When influenza is recognized as a cardiovascular trigger, influenza vaccination becomes an actionable prevention measure rather than a solely respiratory intervention.

## Figures and Tables

**Table 1:** Results and interpretation for influenza vaccination and high-dose influenza vaccine studies discussed in this review.

	Clinical Question/Comparator	Main Cardiovascular or Hospitalization Results	Interpretation
IAMI [23]	Influenza vaccination versus placebo after myocardial infarction or high-risk coronary disease.	Primary composite of all-cause death, myocardial infarction, or stent thrombosis: 5.3% versus 7.2%; HR, 0.72 (95% CI, 0.52–0.99). Cardiovascular death: HR, 0.59 (95% CI, 0.39–0.90).	Positive randomized evidence for early vaccination in secondary prevention; trial stopped early during COVID-19, which should temper overinterpretation of precision.
Behrouzi et al. meta-analysis [17]	Influenza vaccination versus placebo or control in randomized trials.	MACE: RR, 0.66 (95% CI, 0.53–0.83). Recent ACS subgroup: RR, 0.55 (95% CI, 0.41–0.75).	Supports cardiovascular benefit of vaccination, with greatest effect in recent ACS and high-risk secondary prevention.
INVESTED [28]	High-dose trivalent versus standard-dose quadrivalent vaccine in patients with recent myocardial infarction or heart failure hospitalization.	Primary composite of all-cause death or cardiopulmonary hospitalization: HR, 1.06 (95% CI, 0.97–1.17); *p* = 0.21.	High-dose vaccine was not superior to standard dose in this high-risk active-comparator trial; vaccination itself remains recommended.
DANFLU-2 [29,30]	High-dose versus standard-dose vaccine in adults aged 65 years or older.	Primary influenza/pneumonia hospitalization: rVE, 5.9% (95% CI, −2.1 to 13.3), neutral. Cardiorespiratory hospitalization: rVE, 5.7% (95% CI, 1.4 to 9.9). Heart failure hospitalization: rVE, 19.5% (95% CI, 3.3 to 33.1). No superiority for MI, stroke, CV death, or MACE.	Suggests incremental benefit for cardiorespiratory and HF hospitalization, but cardiovascular analyses are exploratory in the context of a neutral primary end point.
GALFLU [31]	High-dose versus standard-dose vaccine in community-dwelling adults aged 65 to 79 years.	Primary influenza/pneumonia hospitalization: rVE, 23.7% (95% CI, 6.6 to 37.7). Influenza hospitalization: rVE, 31.8% (95% CI, 5.0 to 51.3).	Positive active-comparator evidence for severe respiratory hospitalization outcomes in older adults.
FLUNITY-HD pooled analyses [32,33]	Individual-level pooled analyses of DANFLU-2 and GALFLU.	Influenza/pneumonia hospitalization: rVE, 8.8% (95% CI, 1.7 to 15.5). Cardiovascular hospitalization: rVE, 6.6% (95% CI, 1.6 to 11.4). Heart failure hospitalization: rVE, 21.3% (95% CI, 7.6 to 33.0).	Strengthens the severe hospitalization and HF-hospitalization signal; absolute effects are modest and depend on baseline risk and season.
PANDA II [27]	Predischarge influenza vaccination strategy versus usual care in hospitalized acute heart failure.	Death or hospital readmission at 12 months: 41.2% versus 47.0%; OR, 0.83 (95% CI, 0.72–0.97).	Supports in-hospital vaccination as an implementable heart failure discharge intervention in a low-coverage setting.

Abbreviations: ACS, acute coronary syndrome; CI, confidence interval; CV, cardiovascular; HD-IIV, high-dose inactivated influenza vaccine; HF, heart failure; HR, hazard ratio; MACE, major adverse cardiovascular events; MI, myocardial infarction; OR, odds ratio; RR, risk ratio; rVE, relative vaccine effectiveness; SD-IIV, standard-dose inactivated influenza vaccine.

**Table 2:** Comparison of guideline and recommendation documents relevant to influenza vaccination in cardiovascular prevention.

	Target Populations	Vaccine Type or Formulation Considerations	Cardiology-Specific Implication
World Health Organization position paper [3]	Older adults, people with chronic health conditions, pregnant women, health workers, and other nationally defined high-risk groups.	Annual seasonal influenza vaccination; product selection is generally adapted to national programs, availability, age, and risk group.	Provides the public-health foundation for identifying cardiovascular patients and older adults as priority groups.
European Society of Cardiology clinical consensus statement [1]	Patients with established cardiovascular disease and other high-risk cardiovascular profiles, with emphasis on prevention across cardiovascular care.	Supports clinically appropriate adult vaccines, including influenza vaccination; timing and product choice should follow national/regional guidance.	Places vaccination within cardiovascular prevention and supports assessment of vaccination status during cardiology encounters and transitions of care.
American College of Cardiology concise clinical guidance [2]	Adults with cardiovascular disease, older adults, and patients encountered during hospital admission or outpatient cardiovascular care.	Annual influenza vaccination; cardiology teams should support delivery or referral according to eligibility, contraindications, and local policy.	Frames immunization as a routine component of cardiovascular care pathways and quality improvement.
Spanish/SEMERGEN adult-vaccination recommendations [5]	Older adults and adults with chronic conditions, including cardiovascular disease, with coordination between primary care and specialty care.	Seasonal vaccination, with enhanced-immunogenicity formulations considered in older adults according to Spanish/regional policies and product availability.	Provides an implementation bridge for cardiology-primary care coordination, risk registers, and opportunistic vaccination.

**Table 3:** Practical implementation pathways for influenza vaccination in cardiovascular care.

Care Setting	Trigger Point
ACS discharge	Index hospitalization, discharge checklist, or early post-discharge visit.
Heart failure discharge	Admission for acute or decompensated heart failure, especially before transition home or to rehabilitation.
Outpatient cardiology	Pre-season visits, annual reviews, device clinics, anticoagulation visits, and post-ACS/HF follow-up.
Cardiac rehabilitation	Program intake and seasonal sessions.
Primary care	Annual influenza campaign and chronic-disease reviews.
Nursing homes/long-term care	Pre-season campaign and new-resident admission.
Pharmacy-based pathways	Walk-in seasonal vaccination or cardiology/primary-care referral.

Abbreviations: ACS, acute coronary syndrome; HF, heart failure.

## Data Availability

The original contributions presented in this study are included in the article. Further inquiries can be directed to the corresponding authors.

## Abbreviations

The following abbreviations are used in this manuscript:

MACE	Major Adverse Cardiac Events
